# Symptom-based diagnostic models for common respiratory viral infections: a machine learning and natural language processing study

**DOI:** 10.1016/j.idm.2026.04.006

**Published:** 2026-04-09

**Authors:** Mingqing Xie, Suyi Zhang, Jianyong Shen, Yan Liu, Ye Yao, Peng Zhang, Weibing Wang

**Affiliations:** a Department of Biostatistics, School of Public Health, Fudan University, Shanghai, China; b Huzhou Center for Disease Control and Prevention, Zhejiang, China; c Shanghai Institute of Infectious Disease and Biosecurity, School of Public Health, Fudan University, Shanghai, China; d Key Laboratory of Public Health Safety of Ministry of Education, Fudan University, Shanghai, China

**Keywords:** Machine learning, Respiratory viral infections, Natural language processing, Multiplex pathogen detection, Primary triage

## Abstract

**Aim:**

This study aimed to develop an efficient and cost-saving diagnostic approach using natural language processing and explainable machine learning models.

**Subject and methods:**

11,863 Influenza-like illness cases from Huzhou City, China for four common respiratory viruses were collected: SARS-CoV-2, influenza, respiratory syncytial virus, and adenovirus. Natural language processing techniques were employed to extract and normalize symptom features from unstructured clinical text. Five machine learning algorithms were evaluated using AUC, accuracy, sensitivity, and specificity to select the best-performing model. Subgroup analyses by age, sex, and fever status assessed model robustness, and SHAP values were calculated for interpretability.

**Results:**

Compared with existing diagnostic tools, our model demonstrated higher accuracy and better predictive performance, with AUCs of 0.856 (95% CI: 0.830–0.881) for SARS-CoV-2, 0.737 (95% CI: 0.713–0.760) for Influenza, 0.801 (95% CI: 0.744–0.857) for RSV, and 0.782 (95% CI: 0.748–0.816) for adenovirus, showing particularly high capability for SARS-CoV-2 and RSV. Subgroup analyses showed particularly excellent discriminative accuracy in pediatric or afebrile patients.

**Conclusions:**

This study demonstrates the feasibility of integrating natural language processing and machine learning techniques for identification of respiratory viruses based solely on symptoms, and offers a low-cost and efficient alternative to PCR testing, which can reduce reliance on resource-intensive testing and enhance early detection in clinical practice. This approach can support early screening and resource allocation in both clinical and public health settings.

## 1 Background

Respiratory viral infections pose a significant public health threat and substantial disease burden, particularly to young children and older adults (Li et al., 2022; Shi et al., 2020). Recent evidence also suggests that the pandemic has altered seasonal patterns and epidemiological features of non-SARS-CoV-2 respiratory viruses (Eden et al., 2022; Groves et al., 2021; Leuzinger et al., 2020; Ye & Wang, 2022). Concerns have mounted over the concurrent emergence of influenza, respiratory syncytial virus (RSV), and SARS-CoV-2, a situation sometimes termed a “tripledemic” (Maltezou et al., 2023; Mostafa et al., 2024). These common respiratory viruses exhibit a degree of overlap in their clinical presentations (Covaci et al., 2024; Gu et al., 2024)and fast-tracking detection can be challenging due to these nonspecific symptoms. Despite the high accuracy of polymerase chain reaction (PCR) testing (Abels et al., 2001), its high testing cost remains a significant barrier for individuals in resource-limited settings (Mai & Krauthammer, 2017). In the post-pandemic era, the co-prevalence of multiple respiratory viruses has given rise to an alarming surge in the prevalence of respiratory illnesses (Chow et al., 2023; Zhao et al., 2024). The subsequent increase in detection testing has further compounded the burdens faced by healthcare systems. Misdiagnosis leads to suboptimal patient outcomes, resulting in unnecessary medical expenditures (Fitzpatrick et al., 2021). Consequently, the introduction of rapid and cost-saving screening methods is vital for enhancing the quality of medical care and reducing the economic burden on healthcare systems (Sinsky et al., 2016).

Machine learning has shown great promise in analyzing health-related data at scale (Epelde, 2024). Previous research on machine learning for respiratory tract infections have primarily centered on forecasting prognosis and risk stratification (Domínguez-Olmedo et al., 2021; Fenta et al., 2024; Goto et al., 2019; Huang et al., 2022, 2023), while studies specifically focusing on the etiological diagnosis of respiratory infections remain scarce (Mc Cord-De Iaco et al., 2023; Zhou et al., 2024).

However, the need for laboratory results in most existing models could result in longer waiting times and extra burden for healthcare systems. Electronic health records (EHRs) contain valuable information that could improve model discrimination, but this potential is often untapped due to the challenges of processing unstructured clinical text (Meystre & Haug, 2005). Recent studies have highlighted the potential of natural language processing (NLP) and large language models in infectious disease forecasting and diagnosis (Du et al., 2025; Omar et al., 2024). Building on these advances, this limitation can be addressed through the integration of NLP, which enables the extraction of meaningful features from unstructured textual data. Roquette et al. highlighted that incorporating unstructured textual data can significantly enhance model performance compared to using structured data alone (Roquette et al., 2020).

In light of these findings, we aimed to develop a machine learning classifier that could promptly detect individuals with a low risk of a specific respiratory virus based on their symptoms. Preliminary screening is crucial for identifying individuals at high risk of infection, so that accurate PCR testing can be conducted in an economical and targeted manner. This approach may be particularly applicable in resource-limited settings, where intensive diagnostics and treatments should be directed toward those most likely to benefit, thereby maximizing efficiency and minimizing unnecessary healthcare expenditures. This general model can serve as a valuable tool to assist professionals in making clinical decisions, which has the potential to optimize medical resource allocation in under-resourced settings with insufficient testing capacity.

## 2 Methods

### 2.1 Definition of pre- and post-pandemic eras

As China implemented stringent public health and social measures (PHSMs) starting in early 2020, the period from November 2017 to February 2020 was defined as the pre-pandemic era. The post-pandemic era was defined as the period from December 2022 to April 2024, following the relaxation of COVID-19 policies in December 2022, marked by the release of the “Notice on Further Optimizing the COVID-19 Response” outlining 20 prevention and control measures.

### 2.2 Study design and participants

Data were retrospectively accessed for research purposes from 13,221 influenza-like illness (ILI) surveillance cases during the study period, from 01/11/2017 to 02/29/2020, and from 12/04/2022 to 04/29/2024 at two hospitals in Huzhou City, China. The research team had no access to any information that could identify individual participants during or after data collection. Virus prevalence during the pre-pandemic and post-pandemic periods under the same surveillance system in Huzhou has been reported previously by the Huzhou Center for Disease Control and Prevention (Yang et al., 2025) and is therefore not presented separately in detail here. All data were de-identified by the data provider prior to transfer. Considering the limited number of confirmed cases of the other three viruses and the concern about potential interference from the pandemic, sample collection was suspended during the interim period between the pre- and post-pandemic eras (February 2020 to December 2022).

**Fig. 1:**
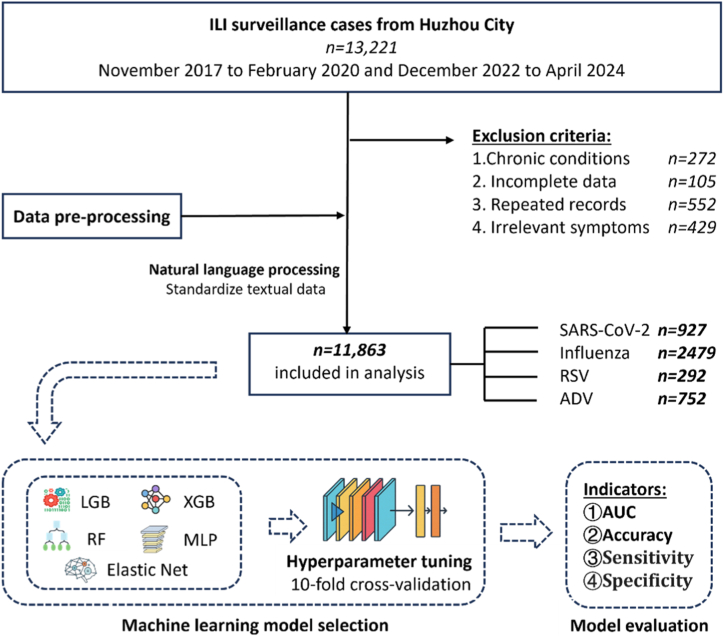
Study flow chart. An illustration of how the diagnosis tool is established via five machine learning algorithms.

Inclusion criteria were as follows: (1) No history of severe underlying conditions such as HIV infection or malignancy. Patients with HIV infection were excluded to reduce potential confounding related to immunocompromised status and to improve cohort homogeneity. (2) Case selection aligned with the sampling guidelines issued by the National Influenza Surveillance Protocol of the National Health Commission of China. Patients who met any of the following criteria were excluded from the study: (1) Chronic diseases such as immunodeficiency, chronic respiratory conditions, liver diseases, malignant tumors, or mental diseases; (2) Repeated records; (3) History of trauma or other non-viral causes of clinical symptoms; (4) Incomplete clinical data. After the removal of unqualified samples, this study comprised a number of 11,863 samples in total (shown in Fig. 1).

### 2.3 Predictors and outcomes

A total of 495 predictive predictors were screened: (1) Demographic characteristics, including age and sex; (2) Unstructured clinical text, including embedded symptom information such as cough, fever, sputum, fatigue, rhinorrhea, diarrhea, nausea, and skin rash, among others; (3) Medical narratives, including past medical records, epidemiological exposure, and history of present illness; (4) Physician-documented diagnoses, including otitis media, acute tonsillitis, and conjunctivitis, among others. Every patient was assigned a main diagnosis, with additional secondary diagnoses recorded when clinically relevant. All diagnoses were collected from community and diagnosed by clinical doctors in structured format, recorded in accordance with the ICD-10 codes (see eTable.1 in Appendix).

The diagnostic outcome was defined as a binary variable indicating infection status based on multiplex PCR testing (RT-PCR) results, served as the dependent variable for model training and evaluation. For each virus, a separate binary classification model was developed. Co-infected cases were retained and labeled as positive in each model. The same labeling strategy was consistently applied in both the training and test sets. Samples for RT-PCR were collected from a variety of sources, including nasopharyngeal and throat swabs, from November 2017 to February 2020 and from December 2022 to April 2024 at two hospitals in Huzhou City, China. The intended prediction time point was defined as the time of first clinical presentation, prior to RT-PCR testing. They were placed in a dedicated non-inactivated sampling tube following collection, stored at 4 °C, and sent to the laboratory within 48 h. Samples were then stored in a −80 °C freezer. In 2024, the collected samples were tested using RT-PCR by the laboratory of Huzhou Center for Disease Control & Prevention, targeting four respiratory viruses: SARS-CoV-2, influenza, RSV, and adenovirus. All samples, including those collected before the COVID-19 pandemic and tested later together with post-pandemic samples, were processed by the same laboratory provider under standardized testing protocols and quality control procedures (Yang et al., 2025). After obtaining each patient's RT-PCR result, we matched it with the corresponding diagnosis records, symptom texts, and clinical history data from the HIS.

### 2.4 Data pre-processing

The study incorporated two formats of indicators: structured data, and unstructured clinical symptom descriptions. For the structured data, categorical features were transformed into binary indicators using one-hot encoding for subsequent modeling. For the unstructured text, we employed a Chinese-language NLP pipeline to extract respiratory symptom information from unstandardized statements recorded in the hospital information system (HIS). Specifically, the text was first segmented using the jiebaR package, and symptom-related terms were identified and mapped to standardized clinical concepts. Semantic normalization was then performed to unify synonymous symptom expressions into standardized terms according to a predefined symptom mapping table (see eTable.2 in Appendix).

The extracted symptom features were subsequently converted into structured variables, primarily as binary indicators representing the presence or absence of each symptom, and were included as candidate predictors in the models.

### 2.5 Model training and performance evaluation

All candidate variables were used to train a set of machine-learning classifiers, including XGBoost, LightGBM, Random Forest, Elastic Net, and Multilayer Perceptron. The five machine learning classifiers were selected based on their widespread use and demonstrated performance in clinical prediction tasks, as supported by prior studies, providing a balance between predictive accuracy, computational efficiency, and interpretability (Breiman, 2001; Esteva et al., 2019; Haug & Drazen, 2023; Li et al., 2021).A 10-fold cross-validation was performed to assess the model's generalization ability and reduce randomness from data division. The data set was split into two subsets, with 80% (n = 9490) used for training and 20% (n = 2373) for internal validation. The internal validation set was not used for model development, tuning, or optimization, and served exclusively for internal performance assessment. A fixed random seed guaranteed reproducibility. To prevent data leakage, no data from the test set was utilized during the training process. Considering class imbalance, we compared four sampling strategies: base, upsampling, downsampling, and SMOTE. A form of grid search was used to perform hyperparameter tuning for each workflow. All model development procedures, including class imbalance handling, feature selection, and hyperparameter tuning using 10-fold cross-validation, were performed exclusively within the training set. The test set was held out and used only for final model evaluation. This method leveraged the Race-Aggregated ANOVA to efficiently explore and optimize hyperparameters across multiple models. (See eMethods in Appendix).

Model performance was primarily evaluated by the area under the receiver operating characteristic curve (AUC), and the model with the highest average AUC was selected. A stepwise feature selection approach was applied, retaining variables that contributed most to AUC improvement. Based on comprehensive expert consensus, variables with no clinical significance were also dropped. Finally, 34 variables were retained. Details of the 34 predictors included in the final models are provided in eTable.8.

Several indicators were used to evaluate the final model's performance, including accuracy, sensitivity, specificity, and AUC. We calculated Youden's J index to determine the optimal classification threshold for the model. As a visual representation of the model's predictive performance, we ultimately plotted the ROC curve and labeled the AUC value of training and test sets. AUC and their 95% confidence intervals were calculated using the DeLong method.

To further evaluate the model's robustness, subgroup analyses were performed based on age, sex, and fever status. These analyses assessed whether the model maintained consistent predictive performance across different patient subpopulations.

Given the black box problems of machine learning models, external interpretability techniques were necessary to understand how predictions were made. SHapley Additive exPlanations (SHAP) values were therefore employed to interpret feature importance and improve transparency of model predictions.

### 2.6 Statistical analysis

The entire NLP and machine learning process was carried out in R software (version 4.4.2). We used the jiebaR package (version 0.11) for data preprocessing, Chinese text segmentation, and keyword extraction.

Categorical variables were presented as numbers and percentages and compared using the χ^2^ test or Fisher's exact test, as appropriate. Continuous variables were expressed as median and interquartile range (IQR), and comparisons between four groups were performed using the Kruskal-Wallis H test. All statistical tests were two-sided, and a *p* value less than 0·05 was considered statistically significant. The type I error rate was set at 0·05.

## 3 Results

### 3.1 Basic patient characteristics

Following the exclusion criteria, 11,863 patients were included in the study (Table 1). The distribution of positive and negative cases for each virus across training and test sets is shown in Appendix eTable.6, and the numbers of patients with single-infections are summarized in eTable.7. Of these patients, patients infected with SARS-CoV-2 were the oldest (29, IQR: 15–46), and those infected with RSV were youngest (4, IQR: 2–10), with a statistically significant difference observed across groups (*p* < 0.001). Fever, sore throat, chills, cough, and sputum were consistently the most prevalent symptoms among patients infected with all four respiratory viruses.

**Table 1:** Baseline characteristics of participants in the study population.

Variables	SARS-CoV-2 (N = 927)	Influenza (N = 2479)	RSV (N = 292)	Adenovirus (N = 752)	Statistics (T/χ2/z)	P-value
Sex (Female), n (%)	537 (58%)	1360 (55%)	177 (61%)	377 (50%)	14.14^b^	P = 0.003∗∗
Age(years), median (IQR)	29(15,46)	19(8,33)	4(2,10)	6(3,9)	870.72^a^	P < 0.001∗∗∗
Fever, n (%)	701 (76%)	1139 (46%)	66 (23%)	167 (22%)	561.56^b^	P < 0.001**∗∗∗**
Sore throat, n (%)	295 (32%)	448 (18%)	14 (4.8%)	40 (5.3%)	237.34^b^	P < 0.001**∗∗∗**
Chills, n (%)	209 (23%)	291 (12%)	9 (3.1%)	13 (1.7%)	198.47^b^	P < 0.001**∗∗∗**
Cough, n (%)	482 (52%)	813 (33%)	38 (13%)	81 (11%)	376.47^b^	P < 0.001**∗∗∗**
Sputum, n (%)	231 (25%)	365 (15%)	15 (5.1%)	12 (1.6%)	208.03^b^	P < 0.001**∗∗∗**
Rhinorrhea, n (%)	176 (19%)	311 (13%)	12 (4.1%)	11 (1.5%)	144.24^b^	P < 0.001**∗∗∗**
Dizziness, n (%)	102 (11%)	213 (8.6%)	2 (0.7%)	18 (2.4%)	68.23^b^,	P < 0.001**∗∗∗**
Headache, n (%)	205 (22%)	281 (11%)	5 (1.7%)	25 (3.3%)	178.23^b^	P < 0.001**∗∗∗**
Myalgia, n (%)	132 (14%)	240 (9.7%)	8 (2.7%)	9 (1.2%)	104.70^b^	P < 0.001**∗∗∗**
Diarrhea, n (%)	59 (6.4%)	53 (2.1%)	13 (4.5%)	18 (2.4%)	41.88^b^	P < 0.001**∗∗∗**
Pneumonia, n (%)	115 (12%)	142 (5.7%)	20 (6.8%)	33 (4.4%)	55.90^b^	P < 0.001**∗∗∗**
Fatigue, n (%)	84 (9.1%)	146 (5.9%)	13 (4.5%)	15 (2.0%)	39.00^b^	P < 0.001**∗∗∗**
upper respiratory symptoms, n (%)	280 (30%)	1171 (47%)	91 (31%)	243 (32%)	119.03^b^	P < 0.001**∗∗∗**
Acute tonsillitis, n (%)	33 (3.6%)	99 (4.0%)	34 (12%)	149 (20%)	248.00^b^	P < 0.001**∗∗∗**
Chest tightness, n (%)	70 (7.6%)	42 (1.7%)	1 (0.3%)	4 (0.5%)	115.07^b^	P < 0.001**∗∗∗**
Abdominal pain, n (%)	41 (4.4%)	41 (1.7%)	2 (0.7%)	6 (0.8%)	36.98^b^	P < 0.001**∗∗∗**
Acute bronchitis, n (%)	25 (2.7%)	237 (9.6%)	62 (21%)	83 (11%)	101.48^b^	P < 0.001**∗∗∗**
Nausea, n (%)	66 (7.1%)	52 (2.1%)	1 (0.3%)	9 (1.2%)	79.43^b^	P < 0.001**∗∗∗**
Vomiting, n (%)	71 (7.7%)	55 (2.2%)	0 (0%)	11 (1.5%)	87.18^b^	P < 0.001**∗∗∗**
Chest pain, n (%)	21 (2.3%)	23 (0.9%)	0 (0%)	2 (0.3%)	21.40^b^	P < 0.001**∗∗∗**
Acute pharyngitis, n (%)	40 (4.3%)	188 (7.6%)	10 (3.4%)	94 (13%)	47.89^b^	P < 0.001**∗∗∗**
Skin rash, n (%)	6 (0.6%)	20 (0.8%)	5 (1.7%)	7 (0.9%)	0.84^c^	P = 0.423
Stomatitis, n (%)	1 (0.1%)	6 (0.2%)	0 (0%)	10 (1.3%)	3.29^c^	P < 0.001**∗∗∗**
Otitis media, n (%)	2 (0.2%)	3 (0.1%)	2 (0.7%)	6 (0.8%)	2.54^c^	P = 0.011**∗**
Convulsions, n (%)	4 (0.4%)	3 (0.1%)	0 (0%)	1 (0.1%)	1.04^c^	P = 0.311
Acute laryngitis, n (%)	5 (0.5%)	25 (1.0%)	5 (1.7%)	8 (1.1%)	1.04^c^	P = 0.324
Conjunctivitis, n (%)	3 (0.3%)	8 (0.3%)	0 (0%)	7 (0.9%)	1.50^c^	P = 0.130
Urinary tract infection, n (%)	5 (0.5%)	5 (0.2%)	0 (0%)	1 (0.1%)	1.04^c^	P = 0.302
Sepsis, n (%)	0 (0%)	0 (0%)	0 (0%)	3 (0.4%)	2.58 c	P = 0.010 **∗∗**
Enteroviral vesicular pharyngitis, n (%)	1 (0.1%)	0 (0%)	0 (0%)	4 (0.5%)	2.81^c^	P = 0.005**∗∗**
Hand, foot and mouth disease, n (%)	1 (0.1%)	7 (0.3%)	2 (0.7%)	1 (0.1%)	1.04^c^	P = 0.361
Cellulitis, n (%)	1 (0.1%)	3 (0.1%)	0 (0%)	0 (0%)	1.04^c^	P = 0.322

Data are presented as n (%) for categorical variables and as median (IQR) for continuous variables.

∗: p < 0.05, ∗∗: p < 0.01, ∗∗∗: p < 0.001.

a Kruskal-Wallis H test.

b chi-squared test.

c Fisher exact test.

Each virus modeled in this study exhibited unique characteristics. More than two-thirds of patients infected with SARS-CoV-2 had fever (76%), but relatively few reported Acute bronchitis (2.7%). Patients with SARS-CoV-2 infections tended to have sore throat (32%) and acute upper respiratory infection (30%) than patients with other viruses (*p* < 0.001). Adenovirus infection was associated with the highest proportions of acute pharyngitis (13%) and acute tonsillitis (12%) among all viral groups (p < 0.001). In contrast, acute bronchitis was observed in 21% of RSV cases (p < 0.001). In addition, the absence of certain symptoms, such as vomiting (0%) in the RSV group, may serve as a potential indicator for differential diagnosis.

### 3.2 Model evaluation

**Fig. 2:**
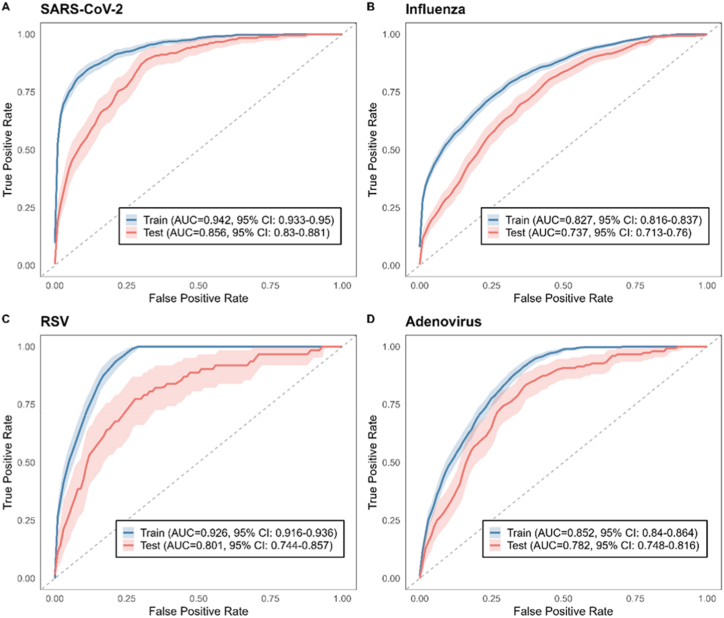
ROC curves of training and test sets for SARS-CoV-2 (a), influenza (b), RSV (c), and adeno**virus (d).** The consistently high AUC values (all >0.7) demonstrate excellent model discrimination. The light band around the curve represents pointwise 95% confidence interval.

To measure the performance among multiple machine learning models, we plotted their ROC curves and identified the best-performing model according to AUC (see eFig. 1 in Appendix). As shown in Fig. 2, our models demonstrated strong and reliable performance in distinguishing among respiratory virus infections. In the test set, the AUCs were 0.856 (95% CI: 0.83–0.881) for SARS-CoV-2, 0.737 (95% CI: 0.713–0.76) for Influenza, 0.801 (95% CI: 0.744–0.857) for RSV, and 0.782 (95% CI: 0.748–0.816) for adenovirus. Although the models for Influenza and adenovirus did not exceed the 0.8 threshold, their performance was still notably better than those reported in comparable studies, highlighting the overall robustness of our approach. An upsampling strategy was employed to address the class imbalance in the RSV and adenovirus datasets.

In addition to AUC, our models also achieved favorable performance in terms of accuracy, sensitivity, and specificity: for SARS-CoV-2, the accuracy was 0.726, with a sensitivity of 0.836 and specificity of 0.717; for Influenza, accuracy reached 0.634, sensitivity 0.730, and specificity 0.609. The RSV model achieved the highest accuracy (0.803) and specificity (0.807), though sensitivity was relatively lower at 0.645. For adenovirus, the model yielded an accuracy of 0.657, sensitivity of 0.822, and specificity of 0.646.

The relatively lower AUC for Influenza may be due to the heterogeneity of influenza subtypes, which complicates feature learning and impairs classification accuracy. For adenovirus, the limited number of positive cases likely contributed to reduced model performance, as the low prevalence of adenovirus in the study population restricted the ability to generalize from training data. In contrast, the models for SARS-CoV-2 and RSV achieved AUCs above 0.8, indicating excellent diagnostic capacity and strong generalization. These findings collectively support the applicability of our models in accurately identifying a range of respiratory viral infections, even under real-world data constraints.

Table 2 presented additional performance metrics of the models. The hyperparameter search space was detailed in Appendix eTable.3. The optimal parameters can be found in Appendix eTable.4. To assess the potential impact of co-infection on model performance, we conducted a sensitivity analysis by excluding patients with multiple viral infections. The results were largely consistent with those from the primary analysis, with only minimal changes in AUC values across all four viruses, suggesting that co-infection did not materially affect model discrimination. Detailed results are presented in Appendix eTable.5 and eFig. 2.

**Table 2:** Model Performance Evaluation for Optimal Classifiers of SARS-CoV-2, influenza, RSV, and adenovirus.

Virus	Model	AUC	Accuracy	Sensitivity	Specificity
SARS-CoV-2	XGBoost	0.86	0.73	0.83	0.72
Influenza	LightGBM	0.74	0.63	0.73	0.61
RSV	XGBoost	0.80	0.80	0.65	0.81
Adenovirus	XGBoost	0.78	0.66	0.82	0.65

The closer the AUC, Accuracy, Sensitivity, and Specificity values are to 1, the better the model's performance. After hyperparameter tuning, the optimal model for influenza was LightGBM, while XGBoost was identified as the best-performing model for RSV, adenovirus, and SARS-CoV-2.

### 3.3 Subgroup analyses

**Fig. 3:**
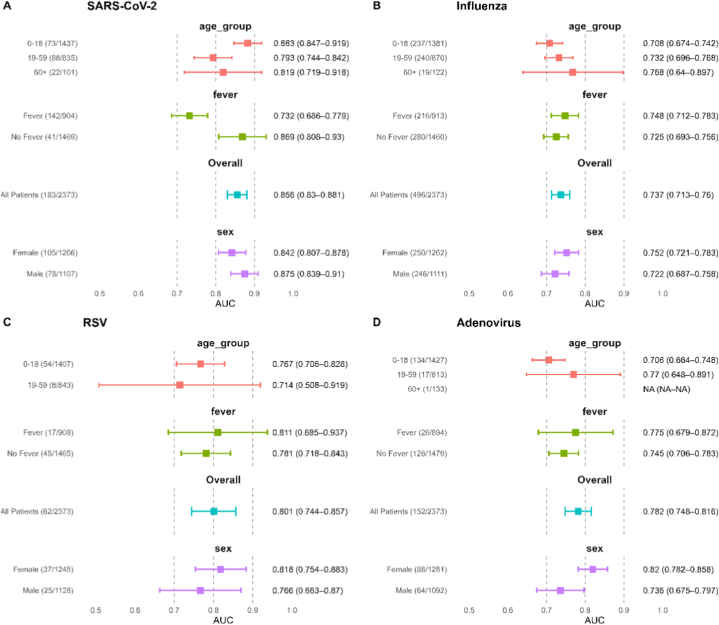
Forest plots for SARS-CoV-2 (a), Influenza (b), RSV (c), and Adeno**virus (d).** Subgroup sample sizes are shown in brackets as n/N, where n denotes the number of positive cases and N denotes the total number of cases in the test set. AUC estimates for the ≥60 age group were not available for RSV and adenovirus due to insufficient positive samples. (Note: AUC estimates for the ≥60 age group were not available for RSV and adenovirus due to insufficient positive samples.)

Fig. 3 shows the subpopulation-specific performance of diagnostic models for SARS-CoV-2, influenza, RSV, and adenovirus across age, sex, and fever status. Overall performance across all patients is also shown for reference. Subgroup analyses for RSV and adenovirus showed greater variability and wider confidence intervals compared to SARS-CoV-2 and Influenza, possibly due to the smaller number of positive cases available for training and evaluation. Performance in the 0–18 age group was consistently high, with AUCs of 0.883 (95% CI: 0.847–0.919) for SARS-CoV-2, 0.708 (0.674–0.742) for Influenza, 0.767 (0.706–0.828) for RSV, and 0.706 (0.664–0.748) for adenovirus. This may reflect the age-related distribution pattern that respiratory viruses are more prevalent in infants and adolescents.

Results stratified by sex were relatively stable, with AUCs similar to those observed in the full population. For instance, for SARS-CoV-2, the AUCs were 0.842 (0.807–0.878) for females and 0.875 (0.839–0.91) for males, compared to 0.856 (0.83–0.881) overall.

Fever status appeared to affect model discrimination. Model performance was generally higher in the non-fever subgroup across multiple pathogens. In the SARS-CoV-2 model, the AUC was 0.869 (0.808–0.93) in patients without fever, compared to 0.732 (0.686–0.779) in those with fever. This finding highlights the potential diagnostic challenges posed by typical symptoms like fever, which may reduce discriminative ability.

### 3.4 Model interpretation using SHAP

**Fig. 4:**
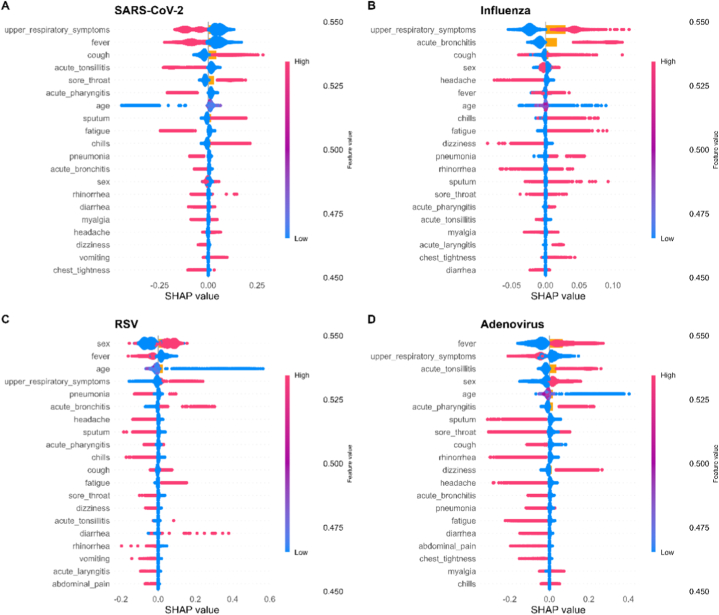
Attributes of characteristic**s in SHAP.** Red spots indicate the presence of a symptom, while blue indicate its absence. Points located to the right of the vertical line indicate a positive contribution to predicting the specific virus, whereas points to the left indicate a negative impact.

Fig. 4 shows the top 20 important features in our models based on SHAP values. Age, upper respiratory symptoms, and fever emerged as top-ranking predictors in all four viruses. Notably, older age was correlated with an increased risk of SARS-CoV-2 infection, whereas younger age strongly predicted RSV infection, consistent with the known age-related susceptibility profiles of these viruses. For example, infants under six months are particularly vulnerable to RSV infections.

Among all viruses included in our models, identical characteristics revealed varying importance. For SARS-CoV-2, acute tonsillitis and age showed substantial impact, suggesting these variables are particularly informative in distinguishing COVID-19 cases. Additionally, acute bronchitis was positively associated with RSV detection, which is consistent with previous studies reporting lower respiratory tract involvement in RSV infections. In the adenovirus model, the absence of sputum, sore throat, and headache demonstrated a higher possibility of infection. For influenza, chills and fatigue contributed significantly.

## 4 Discussion

Globally, respiratory viral infections place a noticeable financial strain on healthcare providers and insurers, underscoring the need for effective prevention (Chen & Guestrin, 2016). The leading contributors include SARS-CoV-2, influenza, and RSV (Grace et al., 2023; Tempia et al., 2019). Accordingly, this study focused on these three major viruses, along with adenovirus, as representative pathogens for investigating common respiratory viral infections. However, earlier detection of respiratory viral infections can be challenging. Consequently, exploring the specific factors related to the diagnosis of common acute respiratory infection is clinically significant. To address this knowledge gap, our study has designed a resource-efficient method for the quick screening of four common respiratory viral infections with the machine learning framework based on the retrospective data collected at two hospitals in Huzhou City, China. Our findings indicate that this approach can function as a supplementary tool for clinicians to achieve timely detection and develop effective treatment strategies. As the model relies solely on clinical symptoms and requires no laboratory testing, it holds strong potential for application in low-resource settings.

Our models exhibited notable predictive capabilities, achieving higher AUC, accuracy, sensitivity, and specificity compared to the baseline. The findings highlight the robustness and clinical viability of our approach. Compared with previous models, our main advantages lie in the integration of machine learning, NLP techniques and efficient hyperparameter tuning algorithms. Our dataset exhibited a class imbalance problem, with substantially fewer positive cases of the target virus among samples. In order to obtain the most appropriate data preprocessing method, five machine learning models were considered, each combined with the four data balancing methods, resulting in a total of 20 workflows. Recent research indicates that machine learning techniques applied in medical decision-making have achieved notable success across diverse diseases and clinical tasks (Epelde, 2024), a trend that is further supported by our findings, which demonstrate strong model performance in distinguishing respiratory viral infections. Although reducing the number of predictors may improve model simplicity and facilitate clinical implementation, our sensitivity analysis (see eFig. 4 in Appendix) suggested that substantial reduction (e.g., to 10 predictors) may lead to model instability and potential overfitting, particularly in the presence of limited sample size and class imbalance.

To evaluate the applicability of our diagnostic model across different clinical populations, we conducted subgroup analyses based on three key variables: age, sex, and fever. For each subgroup, we calculated the AUC along with its 95% confidence interval. This allowed us to assess whether the model's predictive performance varied among different demographic or risk strata. The findings provided insight into the generalizability of the model and help identify specific populations in which the model may perform particularly well or require further calibration.

The model's findings demonstrate strong stability and generalization ability, suggesting that it can serve as an effective early screening tool across various medical environments. First, RSV continues to impose a substantial health burden, particularly in affecting older adults and populations in economically disadvantaged (Du et al., 2023; Nyawanda et al., 2023). Most current models are tailored specifically for pediatric populations (Tozzi et al., 2020), while our model is designed to accommodate individuals across all age groups, enhancing its applicability in diverse healthcare environments.

Second, while many existing ML-based triage models have demonstrated strong performance using laboratory data, our approach focuses exclusively on symptom clusters derived from non-laboratory sources. This allows for early identification and triage of suspected cases without the delays associated with testing. To fully harness symptom data, we further employed natural language processing techniques to capture predictive features from physician notes in the electronic health records. Although such data contain rich symptom information that can serve as strong predictors of infection, they remain underutilized in existing triage models (Tso et al., 2022). By incorporating this routinely available information, our model supports rapid, accessible screening.

Third, our model is built on a large and well-structured dataset of 11,863 patient records and is designed to simultaneously address multiple common respiratory viruses. Compared to models that targeted a single pathogen as in many prior studies (Canas et al., 2021), our multi-pathogen framework offers a more comprehensive and efficient alternative to traditional risk scores or targeted testing strategies. These features improve model robustness and support broader clinical applicability, especially for optimizing resource allocation.

However, several limitations of the present study should be acknowledged. Firstly, the study is constrained by the inadequate number of positive samples for low-prevalence viruses, such as adenovirus, which contributed to the relatively low AUC. Future studies with a longer surveillance period and a larger number of confirmed cases are needed to improve the robustness of the findings. Secondly, the data collection occurred during the COVID-19 pandemic, which may introduce potential confounding factors. However, this also provides a unique opportunity to analyze shifts in disease trends observed pre- and post-pandemic. In addition, as the symptom data provided to our research team were de-identified by the data provider and detailed temporal information was masked for data security reasons, we were unable to further compare symptom distributions between the pre- and post-pandemic periods. Thirdly, variations in healthcare-seeking behavior and differences in medical resource accessibility across regions are likely to undermine the objectivity of data measurement. To that end, future studies could explore the generalizability of this diagnostic tool across diverse healthcare settings, highlighting disparities in both economic development and spatial distribution. Fourthly, although the overall sample size was relatively large, all data were collected from only two hospitals within a single city, which may limit the representativeness of the study population and constrain the generalizability of the model to other healthcare settings. Therefore, the present model should be considered internally validated, and further external validation in broader geographic regions and more diverse clinical settings is warranted before wider application. Furthermore, influenza was modeled as a single aggregated category because routine clinical testing did not consistently distinguish specific subtypes or lineages, and further stratification would have resulted in limited sample sizes for stable analysis. This approach may have obscured subtype-specific heterogeneity in clinical presentation and seasonality.

## 5 Conclusions

Our work offers a new perspective on the detection of respiratory viruses by integrating machine learning with natural language processing techniques. Given its strong performance, we prove that it’s possible to predict the likelihood of infection with clinical symptom records and cut down on unnecessary detection costs. Our algorithm could be utilized to identify people who are more likely to be infected if it is successfully deployed as a quick, early detection tool in a hospital context. By significantly reducing the range of viral testing, this would reduce the waste of scarce resources. Second, this approach allows related personnel in hospitals to detect potentially contagious individuals at an earlier stage, enabling more timely and tailored medical intervention. Further research could aim to validate the model across diverse geographic settings and assess its practicality through prospective studies.

## CRediT authorship contribution statement

**Mingqing Xie:** Writing – original draft, Software, Methodology. **Suyi Zhang:** Writing – original draft, Investigation, Data curation. **Jianyong Shen:** Investigation, Data curation. **Yan Liu:** Investigation, Data curation. **Ye Yao:** Writing – review & editing, Supervision, Funding acquisition. **Peng Zhang:** Writing – review & editing, Supervision, Funding acquisition. **Weibing Wang:** Writing – review & editing, Supervision, Resources, Investigation, Funding acquisition, Data curation.

## Ethics approval and consent to participate

This study strictly adhered to relevant ethical standards and legal regulations. All procedures were approved by the Ethics Committee of the School of Public Health, Fudan University (Approval Number: IRB#2024-05-1116).

## Clinical trial number

Not applicable.

## Consent for publication

This study uses only de-identified data. No personal identifiers are included in the dataset. Since only de-identified data without personal information were used, obtaining consent for publication is not applicable.

## Code availability

The code that has been used for this work has been uploaded to GitHub and is available at https://github.com/ritaxie/ML-based-diagnostic-models_code.

## Funding

This work was supported by the Shanghai Municipal Science and Technology Major Project (Grant No. ZD2021CY001), the Shanghai New Three-year Action Program of Shanghai Municipality for Strengthening the Construction of Public Health System (2023-2025) [Grant No. GWVI-1, GWVI-11.1-03 and GWVI-11.1-01], the Major Program of National Social Science Foundation of China (No. 22&ZD142), and the National Natural Science Foundation of China [Grant No. 82073612], and Disease Prevention and Control Innovation Team of Zhejiang Province (2026JKP-06). The funder of the study had no role in study design, data collection, data analysis, data interpretation, or writing of the report.

## Declaration of competing interest

The authors declare that they have no known competing financial interests or personal relationships that could have appeared to influence the work reported in this paper.

## Abbreviations

RSV, Respiratory Syncytial Virus; NLP, Natural Language Processing; ILI, Influenza-like illness; ML, Machine Learning.

## References

[bib1] Abels S., Nadal D., Stroehle A., Bossart W. (2001). Reliable detection of respiratory syncytial virus infection in children for adequate hospital infection control management. Journal of Clinical Microbiology.

[bib2] Breiman L. (2001). Random forests. Machine Learning.

[bib3] Canas L.S., Sudre C.H., Capdevila Pujol J., Polidori L., Murray B., Molteni E., Graham M.S., Klaser K., Antonelli M., Berry S., Davies R., Nguyen L.H., Drew D.A., Wolf J., Chan A.T., Spector T., Steves C.J., Ourselin S., Modat M. (2021). Early detection of COVID-19 in the UK using self-reported symptoms: A large-scale, prospective, epidemiological surveillance study. The Lancet Digital Health.

[bib4] Chen T., Guestrin C. (2016). Proceedings of the 22nd acm sigkdd international conference on knowledge discovery and data mining.

[bib5] Chow E.J., Uyeki T.M., Chu H.Y. (2023). The effects of the COVID-19 pandemic on community respiratory virus activity. Nature Reviews Microbiology.

[bib6] Covaci S., Filimon C., Craiu M. (2024). Exploring the clinical characteristics and outcomes of rhinovirus infection in hospitalized children compared with other respiratory viruses. Children.

[bib7] Domínguez-Olmedo J.L., Gragera-Martínez Á., Mata J., Pachón Álvarez V. (2021). Machine learning applied to clinical laboratory data in Spain for COVID-19 outcome prediction: Model development and validation. Journal of Medical Internet Research.

[bib8] Du Y., Yan R., Wu X., Zhang X., Chen C., Jiang D., Yang M., Cao K., Chen M., You Y., Zhou W., Chen D., Xu G., Yang S. (2023). Global burden and trends of respiratory syncytial virus infection across different age groups from 1990 to 2019: A systematic analysis of the global Burden of Disease 2019 study. International Journal of Infectious Diseases: IJID: official publication of the International Society for Infectious Diseases.

[bib9] Du H., Zhao Y., Zhao J., Xu S., Lin X., Chen Y., Gardner L.M., Yang H. (2025). Advancing real-time infectious disease forecasting using large language models. Nature Computational Science.

[bib10] Eden J.S., Sikazwe C., Xie R., Deng Y.M., Sullivan S.G., Michie A., Levy A., Cutmore E., Blyth C.C., Britton P.N., Crawford N., Dong X., Dwyer D.E., Edwards K.M., Horsburgh B.A., Foley D., Kennedy K., Minney-Smith C., Speers D., Tulloch R.L., Australian RSV study group (2022). Off-season RSV epidemics in Australia after easing of COVID-19 restrictions. Nature Communications.

[bib11] Epelde F. (2024). How AI could help us in the epidemiology and diagnosis of acute respiratory infections?. Pathogens.

[bib12] Esteva A., Robicquet A., Ramsundar B., Kuleshov V., DePristo M., Chou K., Cui C., Corrado G., Thrun S., Dean J. (2019). A guide to deep learning in healthcare. Nature Medicine.

[bib13] Fenta H.M., Zewotir T.T., Naidoo S., Naidoo R.N., Mwambi H. (2024). Factors of acute respiratory infection among under-five children across sub-saharan African countries using machine learning approaches. Scientific Reports.

[bib14] Fitzpatrick T., Malcolm W., McMenamin J., Reynolds A., Guttmann A., Hardelid P. (2021). Community-based antibiotic prescribing attributable to respiratory syncytial virus and other common respiratory viruses in young children: A population-based time-series study of Scottish children. Clinical Infectious Diseases: An Official Publication of the Infectious Diseases Society of America.

[bib15] Goto T., Camargo C.A., Faridi M.K., Freishtat R.J., Hasegawa K. (2019). Machine learning-based prediction of clinical outcomes for children during emergency department triage. JAMA Network Open.

[bib16] Grace M., Colosia A., Wolowacz S., Panozzo C., Ghaswalla P. (2023). Economic burden of respiratory syncytial virus infection in adults: A systematic literature review. Journal of Medical Economics.

[bib17] Groves H.E., Piché-Renaud P.P., Peci A., Farrar D.S., Buckrell S., Bancej C., Sevenhuysen C., Campigotto A., Gubbay J.B., Morris S.K. (2021). The impact of the COVID-19 pandemic on influenza, respiratory syncytial virus, and other seasonal respiratory virus circulation in Canada: A population-based study. The Lancet Regional Health. Americas.

[bib18] Gu W.X., Xu K., Wang S.J., Deng F., Dai Q.G., Zou X., Shang Q.X., Chen L.L., Xia Y., Dai W.J., Zha J., Ding S.N., He M., Bao C.C. (2024). Zhonghua liu xing bing xue za zhi = Zhonghua liuxingbingxue zazhi.

[bib19] Haug C.J., Drazen J.M. (2023). Artificial intelligence and machine learning in clinical medicine, 2023. New England Journal of Medicine.

[bib20] Huang T., Le D., Yuan L., Xu S., Peng X. (2023). Machine learning for prediction of in-hospital mortality in lung cancer patients admitted to intensive care unit. PLoS One.

[bib21] Huang Y., Talwar A., Lin Y., Aparasu R.R. (2022). Machine learning methods to predict 30-day hospital readmission outcome among US adults with pneumonia: Analysis of the national readmission database. BMC Medical Informatics and Decision Making.

[bib22] Leuzinger K., Roloff T., Gosert R., Sogaard K., Naegele K., Rentsch K., Bingisser R., Nickel C.H., Pargger H., Bassetti S., Bielicki J., Khanna N., Tschudin Sutter S., Widmer A., Hinic V., Battegay M., Egli A., Hirsch H.H. (2020). Epidemiology of severe acute respiratory syndrome coronavirus 2 emergence amidst community-acquired respiratory viruses. Journal of Infectious Diseases.

[bib23] Li Y., Wang X., Blau D.M., Caballero M.T., Feikin D.R., Gill C.J., Madhi S.A., Omer S.B., Simões E.A.F., Campbell H., Pariente A.B., Bardach D., Bassat Q., Casalegno J.S., Chakhunashvili G., Crawford N., Danilenko D., Do L.A.H., Echavarria M., Gentile A., RESCEU investigators (2022). Global, regional, and national disease burden estimates of acute lower respiratory infections due to respiratory syncytial virus in children younger than 5 years in 2019: A systematic analysis. Lancet (London, England).

[bib24] Li Z.J., Zhang H.Y., Ren L.L., Lu Q.B., Ren X., Zhang C.H., Wang Y.F., Lin S.H., Zhang X.A., Li J., Zhao S.W., Yi Z.G., Chen X., Yang Z.S., Meng L., Wang X.H., Liu Y.L., Wang X., Cui A.L., Lai S.J., Chinese Centers for Disease Control and Prevention (CDC) Etiology of Respiratory Infection Surveillance Study Team (2021). Etiological and epidemiological features of acute respiratory infections in China. Nature Communications.

[bib25] Mai M.V., Krauthammer M. (2017). Annual symposium proceedings. AMIA symposium, 2016.

[bib26] Maltezou H.C., Papanikolopoulou A., Vassiliu S., Theodoridou K., Nikolopoulou G., Sipsas N.V. (2023). COVID-19 and respiratory virus co-infections: A systematic review of the literature. Viruses.

[bib27] Mc Cord-De Iaco K.A., Gesualdo F., Pandolfi E., Croci I., Tozzi A.E. (2023). Machine learning clinical decision support systems for surveillance: A case study on pertussis and RSV in children. Frontiers in Pediatrics.

[bib28] Meystre S., Haug P.J. (2005). Automation of a problem list using natural language processing. BMC Medical Informatics and Decision Making.

[bib29] Mostafa H.H., Fall A., Norton J.M., Sachithanandham J., Yunker M., Abdullah O., Hanlon A., Gluck L., Morris C.P., Pekosz A., Klein E.Y. (2024). Respiratory virus disease and outcomes at a large academic medical center in the United States: A retrospective observational study of the early 2023/2024 respiratory viral season. Microbiology Spectrum.

[bib30] Nyawanda B.O., Murunga N., Otieno N.A., Bigogo G., Nyiro J.U., Vodicka E., Bulterys M., Nokes D.J., Munywoki P.K., Emukule G.O. (2023). Estimates of the national burden of respiratory syncytial virus in Kenyan children aged under 5 years, 2010-2018. BMC Medicine.

[bib31] Omar M., Brin D., Glicksberg B., Klang E. (2024). Utilizing natural language processing and large language models in the diagnosis and prediction of infectious diseases: A systematic review. American Journal of Infection Control.

[bib32] Roquette B.P., Nagano H., Marujo E.C., Maiorano A.C. (2020). Prediction of admission in pediatric emergency department with deep neural networks and triage textual data. Neural Networks: The Official Journal of the International Neural Network Society.

[bib33] Shi T., Denouel A., Tietjen A.K., Campbell I., Moran E., Li X., Campbell H., Demont C., Nyawanda B.O., Chu H.Y., Stoszek S.K., Krishnan A., Openshaw P., Falsey A.R., Nair H., RESCEU Investigators (2020). Global disease burden estimates of respiratory syncytial virus-associated acute respiratory infection in older adults in 2015: A systematic review and meta-analysis. Journal of Infectious Diseases.

[bib34] Sinsky C., Colligan L., Li L., Prgomet M., Reynolds S., Goeders L., Westbrook J., Tutty M., Blike G. (2016). Allocation of physician time in ambulatory practice: A time and motion study in 4 specialties. Annals of Internal Medicine.

[bib35] Tempia S., Moyes J., Cohen A.L., Walaza S., Edoka I., McMorrow M.L., Treurnicht F.K., Hellferscee O., Wolter N., von Gottberg A., Nguweneza A., McAnerney J.M., Dawood H., Variava E., Cohen C. (2019). Health and economic burden of influenza-associated illness in South Africa, 2013-2015. Influenza and other respiratory viruses.

[bib36] Tozzi A.E., Gesualdo F., Rizzo C., Carloni E., Russo L., Campagna I., Villani A., Reale A., Concato C., Linardos G., Pandolfi E. (2020). A data driven clinical algorithm for differential diagnosis of pertussis and other respiratory infections in infants. PLoS One.

[bib37] Tso C.F., Lam C., Calvert J., Mao Q. (2022). Machine learning early prediction of respiratory syncytial virus in pediatric hospitalized patients. Frontiers in Pediatrics.

[bib38] Yang M.Y., Liu Y., Zhang S.Y., Wang Q., Liu G.T., Zheng B., Wang X.Y., Zhao D.N., Shen J.Y., Wang W.B. (2025). Epidemiological characteristics of common viral respiratory infections before and after the COVID-19 pandemic in Huzhou, Zhejiang Province. Fudan University Journal of Medical Sciences.

[bib39] Ye Q., Wang D. (2022). Epidemiological changes of common respiratory viruses in children during the COVID-19 pandemic. Journal of Medical Virology.

[bib40] Zhao P., Zhang Y., Wang J., Li Y., Wang Y., Gao Y., Zhao M., Zhao M., Tan H., Tie Y., Feng Z. (2024). Epidemiology of respiratory pathogens in patients with acute respiratory infections during the COVID-19 pandemic and after easing of COVID-19 restrictions. Microbiology Spectrum.

[bib41] Zhou X., Zhang J., Deng X.M., Fu F.M., Wang J.M., Zhang Z.Y., Zhang X.Q., Luo Y.X., Zhang S.Y. (2024). Using random forest and biomarkers for differentiating COVID-19 and Mycoplasma pneumoniae infections. Scientific Reports.

