# Supplementary material for: Symptom-based diagnostic models for common respiratory viral infections: a machine learning and natural language processing study
Source: Infect Dis Model. 2026 Apr 9;11(4):1302–13. doi: 10.1016/j.idm.2026.04.006 (PMC13196312; doi:10.1016/j.idm.2026.04.006)
Supplement: Multimedia component 1 [file mmc1.docx]

**Supplementary**

eMethods 2

eTable 1. ICD-10 Codes used for diagnosis 4

eTable 2. Symptom mapping table for Chinese Clinical Narratives 6

eTable 3. Hyperparameter space explored for machine learning classifiers 7

eTable 4. Optimal hyperparameters for final models 8

eTable 5. AUC comparison with and without co-infected cases 9

eTable 6. Distribution of positive and negative cases in the training and test sets for each virus 10

eTable 7. Number of cases with single-infection for each virus 11

eTable 8. 34 predictors used in the final models 12

eFigure 1. Model performance comparison across five machine learning algorithms 13

eFigure 2. ROC curves of training and test sets for SARS-CoV-2 (a), influenza (b), RSV (c), and adenovirus (d) without co-infected cases 15

eFigure 3. Overview of the modeling workflow 16

eFigure 4. Sensitivity analysis comparing model performance with 10 predictors 17

**eMethods**

1. **Natural Language Processing**

Given that the epidemiological history of the patients and some clinical symptoms of viral infections were documented in the History of Present Illness section during data collection, we decided to employ NLP techniques to separate the descriptions of respiratory clinical symptoms. These technologies hold the potential to markedly decrease both the time and labor involved in data mining, while improving the reliability of extracted data and minimizing human error. NLP can help standardize the extraction process across medical dataset, avoiding issues like information fatigue and variability in performance. To detect and extract vague and non-standard symptom descriptions from the History of Present Illness section and standardized them, we figured out a mapping table to extract every possible description for each eligible patient in the study. These descriptions would be converted to feature words included cough, fever, sore throat, sputum, headache, fatigue, rhinorrhea, vomiting, chest tightness, diarrhea, chills, dizziness, pneumonia, nausea, chest pain, skin rash, and myalgia.

We preprocessed the text by removing special characters, punctuation, and leading whitespace. Any irrelevant or daily vocabulary were specified as stop words and subsequently removed, so that only clinically relevant terms left. The symptom mapping table was used as a named vector, enabling the efficient replacement of original vague terms with standardized terms.

After NLP preprocessing, the text data consists of individual symptom terms, which have been extracted and standardized. These segmented symptom terms will be used in subsequent steps for feature selection and model training, thus improving the predictive accuracy of the model by focusing on clinically relevant information. As supplementary indicators to primary diagnoses, these viral infection symptoms also contribute to the construction of a diagnostic scoring system. This structured approach ensures that each symptom is clearly defined and consistently represented, enabling more effective analysis, supporting comprehensive diagnostic evaluations, and improving model performance.

To enhance reproducibility, the NLP pipeline used in this study has been fully documented and made publicly available. Clinical free-text narratives were processed using the jiebaR package for Chinese word segmentation. Extracted symptom terms were mapped to standardized clinical concepts using a predefined synonym dictionary.

The final set of predictors was constructed by combining structured variables with symptoms extracted from free text. A complete list of the predictors used in the final models, along with their definitions and data sources, is provided in etab.8.

All code used for data processing, NLP implementation, and model development has been made available on GitHub to facilitate transparency and reproducibility.

Natural language processing techniques have the potential to markedly decrease both the time and labor involved in data mining while improving the reliability of extracted data and minimizing human error. The standardized diagnostic terminologies will be used for feature selection and model training as supplementary indicators to primary diagnoses. These will contribute to the construction of a diagnostic scoring system. This structured approach ensures that each symptom is clearly defined and consistently represented, supporting comprehensive diagnostic evaluations and improving model performance.

1. **Feature Selection**

Our feature selection algorithm will filter indicators based on each predictors' relationship with the response outcomes measured using a Receiver Operating Characteristic (ROC) curve. The algorithm follows a stepwise approach, eliminating or adding variables based on their ROC impact. Variables whose exclusion leads to the largest reduction in AUC are considered the most important. This process continues until a subset of variables that maximizes model performance is selected.

We used a 10-fold cross-validation for hyperparameter tuning. In 10-fold cross-validation, the training set is divided into 10 subsets. In each iteration, 9 of these subsets are used for training, while the remaining fold served as the validation set. By calculating the average performance metric (AUC) across folds, the best model and parameters can be identified. When conducting ten-fold cross-validation, we set a stratification parameter to ensure that the distribution of the dependent variables is similar between the training and test sets.

For the selection of optimal parameters and models, five machine learning models were considered, each combined with the four data balancing methods, resulting in a total of 20 workflows.

The 95% confidence intervals (CIs) for AUC were calculated using the DeLong method, as implemented in the pROC package (version 2.3.1) and pROC package (version 1.18.5) in R. This nonparametric approach estimates the variance of the AUC based on U-statistics and is widely used for comparing correlated ROC curves.

For subgroup analyses, AUCs and their corresponding 95% CIs were calculated separately within each subgroup using the same approach.

During the cross-validation process, the algorithm started by evaluating the first three parameter configurations. If any of these configurations outperform the average performance of the first one, further computations can be carried out. However, if all three perform poorly, it can be inferred that subsequent configurations are likely to yield suboptimal results. Thus, there was no need to test them all. This approach effectively reduces computation time and enhances efficiency.

1. **Comprehensive Analysis of Classified Multi-Model**

XGBoost, LightGBM, Random Forest, Elastic Net, and MLP were then trained and tested. We used a 10-fold cross-validation for hyperparameter tuning. The model was evaluated using AUC values.

We set up and a total of 20 workflows, the ideal parameters acquired were at a certain distance from the upper and lower limits of the settings, indicating that the parameter range was appropriate. We believe that there is no need for more statistical testing to prove that the model we ultimately chose outperformed alternative models, as we have already carried out the 10-fold cross-validation on 20 workflows separately. We may safely think that this model is the best, according to the cross-validation results rather than random AUC results.

The tidymodels package (version 1.2.0) was used for constructing our machine learning models. As for the LightGBM, bonsai package (version 0.3.1.9000) was additionally required.

**eTable 1: ICD-10 Codes used for diagnosis**.

All diagnoses were collected from community healthcare settings and recorded in structured format by clinical doctors, following the ICD-10 classification system

| **Diagnosis** | **ICD-10 Codes** |
| --- | --- |
| Cystitis | N30.9 |
| Acute tonsillitis | J03.9, J36, J03.8, J03.0, J03.9 |
| Pneumonia | J12.9, J18.9, J18.0, J22, J15.9, J18.0, J18.0, J98.4 |
| Skin rash | B09, B02.9, B00.9 |
| Acute upper respiratory infections | J06.9, J98.8, J98.9, J39.9, J01.9, J98.7 |
| Diseases of the digestive system | A09.0, A09.9, K59.9, K58.9, I88.0, K52.9, K29.1, K29.2, K29.7, K92.2, K25.9 |
| Cheilitis | K13 |
| Nausea | R11.0 |
| Fever | R50.9, B99, R50.8 |
| Febrile convulsions | R56.0 |
| Fatigue | R53 |
| Pneumonia due to Mycoplasma pneumoniae | J15.7 |
| Cellulitis | L03.9 |
| Abdominal pain | R10.4 |
| Dermatitis | L30.3, L20.9, L23.9 |
| Asthma | J45 |
| Cough | R05 |
| Myalgia | M79.1 |
| Cold | J00, J11.1, J11, J10.8, J10.1 |
| Acute bronchitis | J04.2, J20.8, J20.9, J40 |
| Acute laryngitis | J04.0, J06.0 |
| Acute pharyngitis | J02.9, J02.8, J02.9 |
| Conjunctivitis | H10.3, H10.9, H10.8, H00.0, B30.2 |
| Thyroid disorder | E03.9, Z86.3, E05.9, R94.6, E05.9, E07.9 |
| Neck pain | M54.2 |
| Oral and periodontal diseases | K12.1, K04.0, K05.6, K05.1, B37.0 |
| Pain in joint | M25.5 |
| COPD | J44 |
| Urinary tract infection | N39.0 |
| Sepsis | A41.9 |
| Vomiting | R11 |
| Hand, foot and mouth disease | B08.4 |
| Headache | R51 |
| Dizziness | R42, R55 |
| Chest tightness | R09.8 |
| Chest pain | R07.4, R07.3 |
| Sore throat | R07.0 |
| Mycoplasma infection | A49.3 |
| Otitis media | H66.9 |

**eTable 2: Symptom mapping table for Chinese Clinical Narratives**.

The standardized English symptom terms, their corresponding normalized Chinese terms, and representative original expressions observed in the source texts. To standardize symptom-related features extracted from unstructured clinical narratives, we performed semantic normalization by mapping a wide range of original symptom expressions to unified standardized terms.

| **Standardized Symptom Term** | **Normalized Term** | **Original Term** |
| --- | --- | --- |
| fever | 发热 | 体温, 高热, 热, 发热, 体温升高, 发烧, 温度升高, 体温异常, 异常体温, 发体热, 体温偏高, 感觉发热, 感觉热, 发热感, 发热不退, 持续发热, 间断发热, 高体温, 升温, 热感, 有发烧症状, 体温有点高, 晨起高热, 午后低热, 晚间发热, 夜间发热 |
| sore throat | 咽痛 | 咽部疼痛, 咽部不适, 咽痛, 咽喉痛, 喉咙痛, 咽部红肿, 咽部干燥, 嗓子疼, 喉咙发干, 吞咽痛, 喉咙发炎, 咽部疼, 吞咽不适, 咽红, 喉咙烧灼感, 扁桃体痛, 嗓子发痒, 咽喉发炎, 咽干, 咽部不舒服 |
| sputum | 咳痰 | 痰, 咳痰黄绿, 咳白痰, 咳痰, 咳嗽咳痰, 咳黄痰, 痰多,痰液, 黄痰, 咳出痰, 咳痰不止, 咳痰带血, 痰中带血, 痰黄, 痰白, 咳浓痰 |
| cough | 咳嗽 | 咳嗽, 咳嗽咳痰, 干咳, 剧烈咳嗽, 阵发性咳嗽, 刺激性咳嗽, 持续咳, 嗓子发痒想咳, 轻咳 |
| headache | 头痛 | 头痛, 头疼, 偏头痛, 剧烈头痛, 头胀痛, 颞部痛, 头昏痛 |
| fatigue | 乏力 | 全身乏力, 乏力, 没力气, 虚弱, 身体疲倦, 容易累, 没精神, 全身无力, 感觉累 |
| rhinorrhea | 流涕 | 鼻塞, 流涕, 鼻涕, 打喷嚏, 涕, 鼻涕多, 流鼻水, 鼻子流水, 鼻液, 流清涕, 流浓涕, 打喷嚏带鼻涕, 鼻涕黄, 鼻涕清 |
| vomiting | 呕吐 | 呕吐, 上吐下泻, 吐了, 反胃, 吐东西, 喷射状呕吐, 恶心想吐, 吐酸水, 吐食物, 呕出物 |
| chest tightness | 胸闷 | 喘, 呼吸困难, 胸闷, 喘不上气, 呼吸紧, 胸口堵, 闷气, 气不够, 胸部压迫感, 感觉憋, 呼吸不畅 |
| abdominal pain | 腹痛 | 腹痛, 腹胀, 肚子疼, 肚子痛, 胃疼, 肠子疼, 肠胃不适, 胃部隐痛, 胃痉挛, 下腹胀痛, 腹部不适 |
| diarrhea | 腹泻 | 腹泻, 上吐下泻, 拉肚子, 泻肚, 稀便, 拉稀, 水样便, 大便次数多, 排便频繁, 稀烂便, 肚子拉, 泻肚子 |
| chills | 寒战 | 畏寒, 寒战, 寒颤, 打寒颤, 发抖, 冷战, 浑身发冷, 冷到发抖, 冷颤, 打哆嗦, 发冷 |
| conjunctivitis | 结膜炎 | 红眼, 结膜炎症状, 结膜充血, 流泪, 畏光, 眼部分泌物, 眼痒, 眼痛 |
| otitis media | 中耳炎 | 耳痛, 耳部不适,耳闷, 听力下降, 耳鸣, 中耳炎, 外耳道发炎 |
| dizziness | 头晕 | 头昏, 头昏眼花, 头晕,晕眩, 眼前发黑, 天旋地转, 站不稳, 头脑昏沉, 晕头转向, 眩晕 |
| pneumonia | 肺炎 | 肺炎, 感染性肺炎, 细菌性肺炎, 病毒性肺炎, 双肺感染, 肺部炎症, 胸片异常 |
| nausea | 恶心 | 恶心, 胃部不适, 食欲不振, 想吐, 食欲不佳, 不想吃, 胃里不舒服, 恶心反胃, 吃不下, 进食困难 |
| chest pain | 胸痛 | 胸口闷痛, 胸痛, 心口痛, 胸部疼, 刺痛胸口, 呼吸时痛, 压胸痛, 左胸痛, 右胸痛 |
| skin rash | 皮疹 | 皮疹, 红疹, 起疹子, 出疹, 疹, 身上起疹, 皮肤红点, 红斑, 皮肤瘙痒起疹, 起风疹, 疹子痒 |
| myalgia | 肌肉酸痛 | 肌肉痛, 肌肉酸痛, 全身酸痛, 肌痛, 浑身酸痛, 肌肉疼, 四肢酸痛, 肌肉僵硬, 背酸痛 |
| no exposure history | 无暴露史 | 无疫区旅行史, 无接触史, 无接触人群, 未曾接触, 无疫区旅居, 无相关人员接触, 未外出,无传染病接触, 未去疫情地区, 无疑似接触, 无症状相关接触史 |

**eTable 3: Hyperparameter space explored for machine learning classifiers** **in this study**

The hyperparameter search strategy used during model development. For each machine learning algorithm, relevant hyperparameters were selected for tuning. The "Search Space" column defines the range of values considered for each hyperparameter, while the "Step" column indicates the increment or scale of variation. For parameters using exponential scales (e.g., learning rate, penalty), steps marked with “*10” indicate logarithmic progression. These ranges were chosen based on prior knowledge and empirical guidelines to balance computational efficiency and model performance.

| **Model** | **Hyperparameters** | **Search Space** | **Step** |
| --- | --- | --- | --- |
| XGBoost / LightGBM | tree_depth | [3, 30] | 3 |
|  | n_estimators | [100, 2000] | 100 |
|  | mtry | [0.1, 1] | 0.1 |
|  | min_n | [2, 40] | 2 |
|  | loss_reduction | [0, 10] | 1 |
|  | learn_rate | [1×10 ^−5^, 1×10^−1^] | *10 |
| Random Forest | tree_depth | [3, 30] | 3 |
|  | n_estimators | [100, 2000] | 100 |
|  | mtry | [0.1, 1] | 0.1 |
|  | min_n | [2, 40] | 2 |
| Elastic Net | penalty | [1×10 ^−10^, 1×10^−1^] | *10 |
|  | mixture | [0, 1] | 0.1 |
| Multilayer Perceptron | hidden_units | [1, 100] | 10 |
|  | penalty | [1×10 ^−10^, 1×10^−1^] | *10 |
|  | epochs | [10, 1000] | 100 |

**eTable 4: Optimal hyperparameters for final models**

The hyperparameters that yielded the best performance and were subsequently used in training the final models for predictive analysis.

| **Virus** | **model** | **mtry** | **trees** | **min_n** | **tree_depth** | **learn_rate** | **loss_reduction** |
| --- | --- | --- | --- | --- | --- | --- | --- |
| SARS-CoV-2 | XGBoost | 0.7 | 1071 | 5 | 13 | 0.0108 | 1.37×10^−7^ |
| Influenza | LightGBM | 0.4503 | 1262 | 8 | 10 | 0.0143 | 1.70×10^−10^ |
| RSV | XGBoost | 0.5636 | 1172 | 13 | 15 | 0.0218 | 0.75 |
| Adenovirus | XGBoost | 0.5711 | 1865 | 5 | 7 | 0.0757 | 6.39 |

**eTable 5: AUC comparison with and without co-infected cases**

The primary analysis was conducted using the full dataset, including co-infected cases. The sensitivity analysis was restricted to patients with single-virus infections by excluding all co-infected cases.

| **Virus** | **AUC**  **(including co-infected cases)** | **AUC**  **(excluding co-infected cases)** |
| --- | --- | --- |
| SARS-CoV-2 | 0.86 | 0.89 |
| Influenza | 0.74 | 0.74 |
| RSV | 0.80 | 0.77 |
| Adenovirus | 0.78 | 0.72 |

**eTable 6: Distribution of positive and negative cases in the training and test sets for each virus**

Positive and negative cases were defined based on RT-PCR results for each virus. Each virus was modeled as a separate binary outcome, and counts are reported independently for each virus-specific classification task.

| **Virus** | **Train Positive** | **Train Negative** | **Test Positive** | **Test Negative** |
| --- | --- | --- | --- | --- |
| SARS-CoV-2 | 744 | 8746 | 183 | 2190 |
| Influenza | 1983 | 7507 | 496 | 1877 |
| RSV | 230 | 9260 | 62 | 2311 |
| Adenovirus | 601 | 8889 | 151 | 2222 |

**eTable 7: Number of cases with single-infection for each virus**

Single-infection refers to cases in which only one respiratory virus was detected by RT-PCR, with no concurrent detection of other viruses.

| **Virus** | **Single-infection cases** |
| --- | --- |
| SARS-CoV-2 | 750 |
| Influenza | 2279 |
| RSV | 266 |
| Adenovirus | 735 |

**eTable 8: 34 predictors used in the final models**

This table lists the 34 predictors included in the final models, along with their data definitions. Free-text variables were derived using a Chinese-language NLP pipeline based on jiebaR for word segmentation and a predefined synonym mapping dictionary for normalization.

| **Diagnosis** | **Data source** |
| --- | --- |
| Sex | Demographics characteristics |
| Age | Demographics characteristics |
| Fever | Unstructured clinical text |
| Sore throat | Unstructured clinical text |
| Chills | Unstructured clinical text |
| Cough | Unstructured clinical text |
| Sputum | Unstructured clinical text |
| Rhinorrhea | Unstructured clinical text |
| Chest tightness | Unstructured clinical text |
| Dizziness | Unstructured clinical text |
| Headache | Unstructured clinical text |
| Myalgia | Unstructured clinical text |
| Diarrhea | Unstructured clinical text |
| Pneumonia | Physician-documented diagnoses |
| Skin rash | Unstructured clinical text |
| Upper respiratory symptoms | Physician-documented diagnoses |
| Fatigue | Unstructured clinical text |
| Abdominal pain | Unstructured clinical text |
| Nausea | Unstructured clinical text |
| Vomiting | Unstructured clinical text |
| Stomatitis | Physician-documented diagnoses |
| Otitis media | Physician-documented diagnoses |
| Chest pain | Unstructured clinical text |
| Convulsions | Physician-documented diagnoses |
| Acute laryngitis | Physician-documented diagnoses |
| Acute pharyngitis | Physician-documented diagnoses |
| Conjunctivitis | Physician-documented diagnoses |
| Urinary tract infection | Physician-documented diagnoses |
| Sepsis | Physician-documented diagnoses |
| Enteroviral vesicular pharyngitis | Physician-documented diagnoses |
| Hand foot and mouth disease | Physician-documented diagnoses |
| Acute tonsillitis | Physician-documented diagnoses |
| Acute bronchitis | Physician-documented diagnoses |
| Cellulitis | Physician-documented diagnoses |





**eFigure 1:** **Model performance comparison across five machine learning algorithms**

The ROC curves and corresponding AUC values of training and test sets for SARS-CoV-2 (A), influenza (B), RSV (C), and adenovirus (D). Each curve within the subplot represents the performance of different models (Elastic Net, LightGBM, MLP, Random Forest, and XGBoost). The AUC values indicate the discriminatory power of each model, with higher AUC values reflecting better performance. The results indicated that XGBoost showed the optimal performance in detecting SARS-CoV-2, RSV and adenovirus, while LightGBM was considered more ideal for diagnosing influenza.


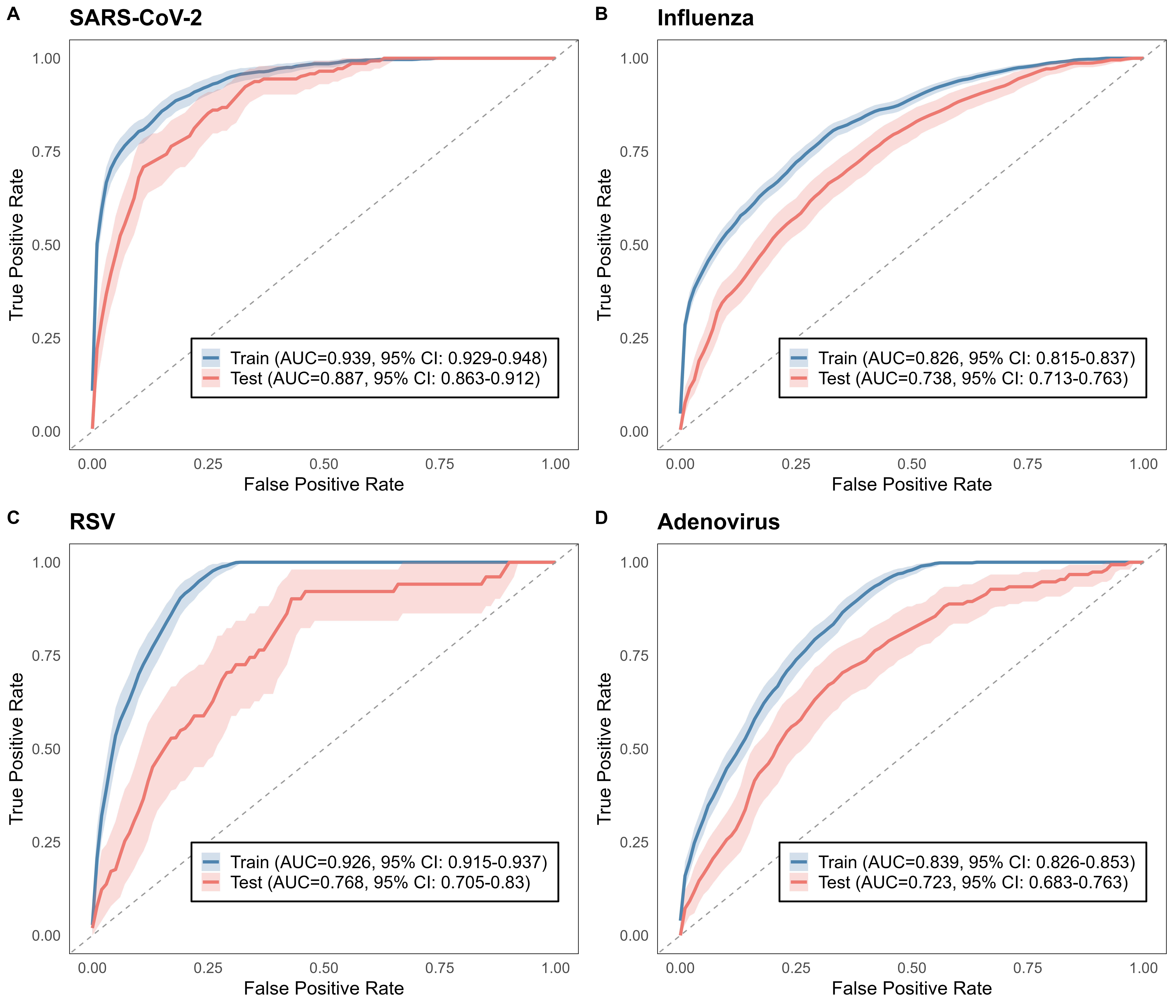


**eFigure 2:** **ROC curves of training and test sets for SARS-CoV-2 (a), influenza (b), RSV (c), and adenovirus (d)** **without co-infected cases**

The ROC curves derived from the single-infection dataset closely resembled those from the full dataset, indicating consistent model performance after excluding co-infected cases.


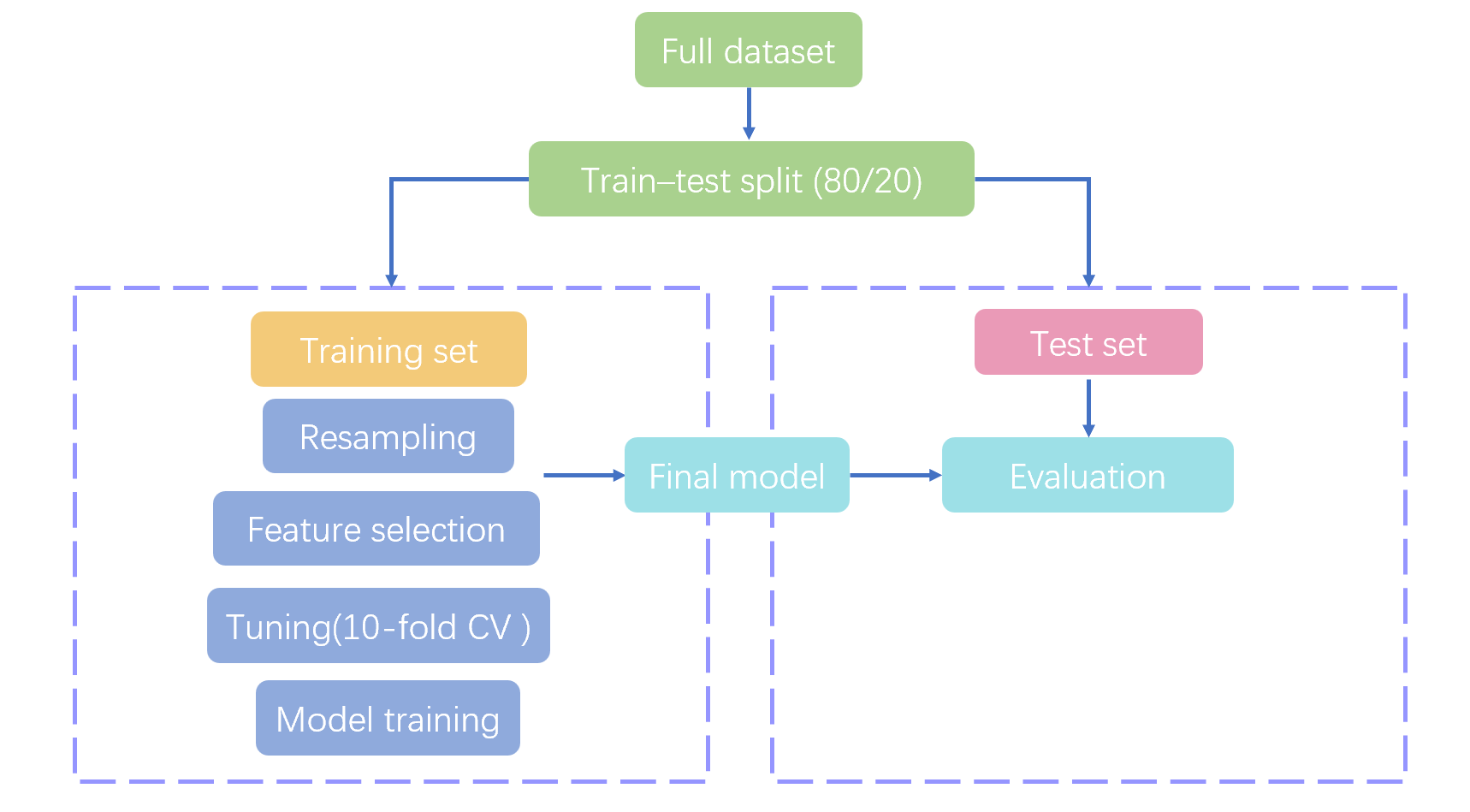


**eFigure 3: Overview of the modeling workflow**

The dataset was first split into training and test sets. All model development procedures, including resampling, feature selection, and hyperparameter tuning, were conducted exclusively within the training set. The test set was held out and used only for final model evaluation.


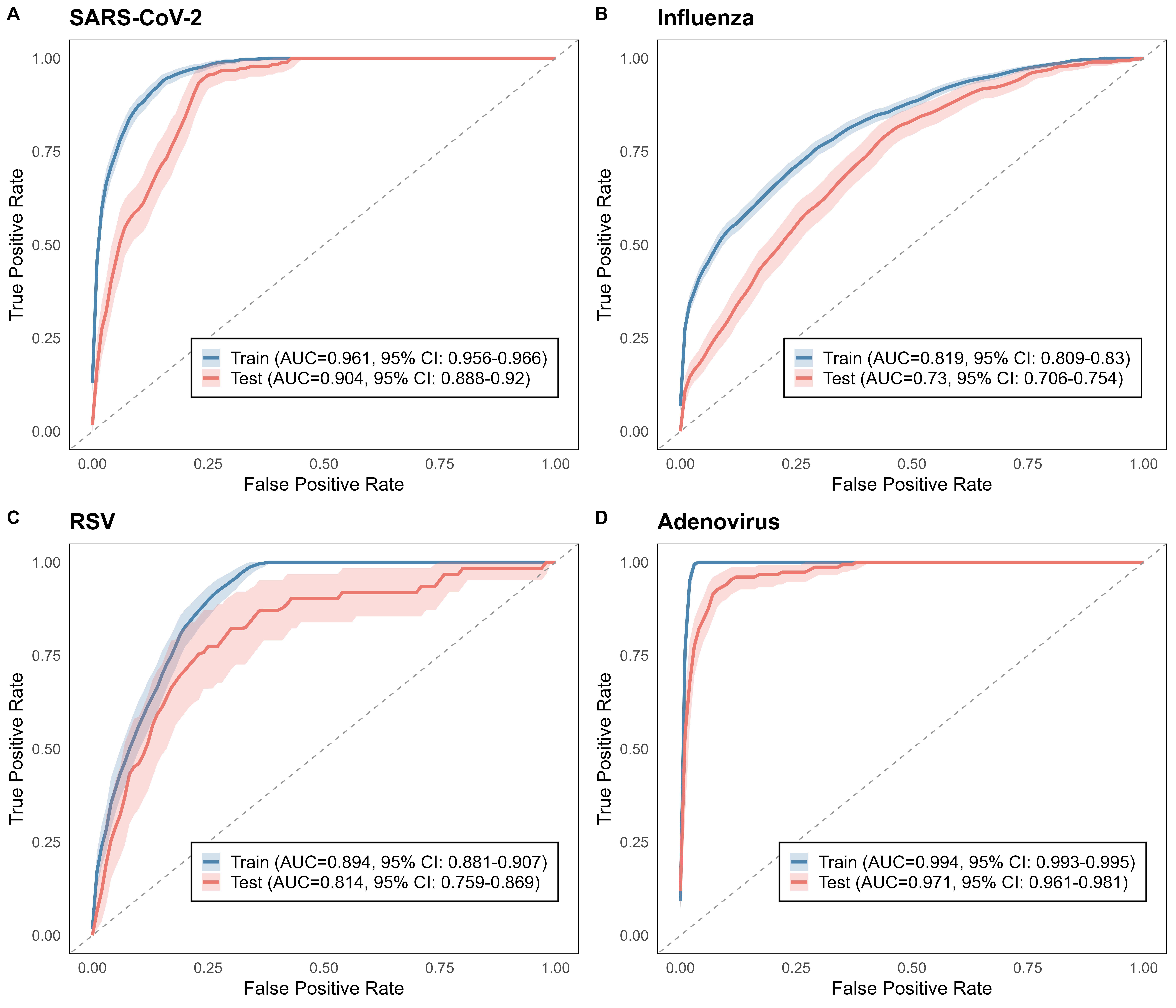


**eFigure 4:** **Sensitivity analysis comparing model performance with 10 predictors**

AUC values of models using the reduced set of 10 predictors across four viruses. Although higher AUCs were observed in some models with fewer predictors, this may reflect model instability and potential overfitting, particularly in the context of limited sample size and class imbalance. The results should therefore be interpreted with caution.
